# The relationship between anxiety and depression symptoms in insomnia patients: a network analysis

**DOI:** 10.1038/s41598-025-09746-w

**Published:** 2025-07-11

**Authors:** Yu Liu, Zirong Zhu, Jiaran Yan, Yuanyuan Wang

**Affiliations:** 1 Department of Neurology, The Air Force Hospital of Northern Theater PLA, Shenyang, 110000 China; 2 Department of Neurology, Xijing Hospital, Fourth Military Medical University, Xi’an, 710032 China; 3 Department of Nursing, The Air Force Hospital of Northern Theater PLA, Shenyang, Liaoning China

**Keywords:** Network analysis, Insomnia, Anxiety, Depression, Bridge symptoms, Neuroscience, Psychology

## Abstract

Anxiety and depressive symptoms are both common in insomnia patients, and they share a critical bidirectional relationship. The available research suggests that transitioning from a disorder-level analysis to a symptom-level analysis may provide a clearer understanding of these relationships. A total of 1571 insomnia patients were enrolled in this study. Sleep quality was assessed using the Pittsburgh Sleep Quality Index (PSQI), anxiety symptoms were evaluated using the Hamilton Rating Scale for Anxiety (HAM-A), and depressive symptoms were evaluated using the Hamilton Rating Scale for Depression (HAM-D). Subsequently, a network analysis was conducted for the statistical analysis. Among the present sample, the prevalences of depression and anxiety were 87.1% and 88.0%, respectively. We found that the strongest regularized partial correlations existed in the network between “C3: Sleep duration” and “C4: Habitual sleep efficiency” (*r* = 0.91). Two other strong regularized partial correlations were observed between “B3: Cognitive disturbance” - “B7: Hopelessness” (*r* = 0.36), “A2: Psychic anxiety” - “B5: Retardation” (*r* = 0.28). In addition, the item “C3: Sleep duration” had the highest strength centrality in the network, followed by “C1: Subjective sleep quality”, “C4: Habitual sleep efficiency”, “A2: Psychic anxiety”, “B1: Anxiety/Somatization”. The findings highlight the crucial role of the strongest regularized partial correlations and bridge symptoms in relation to depression and anxiety. Targeted interventions for clinical control of anxiety and depressive symptoms in insomnia patients are promising.

**Supplementary Information:**

The online version contains supplementary material available at 10.1038/s41598-025-09746-w.

## **Introduction**

Approximately one-third of adults worldwide currently suffer from insomnia^1^. Insomnia is characterized by sleep disorders associated with daytime complaints such as sleepiness, daytime fatigue, low mood, somatic symptoms (e.g., headaches or body aches), compromised cognitive or occupational function, concerns about sleep, or dissatisfaction with sleep^2^. Currently, two main methods are used to treat insomnia: cognitive behavioral therapy for insomnia (CBT-I) and pharmacological treatments. However, despite these treatment options, there are still hundreds of millions of patients with ongoing insomnia^3^. In addition to insomnia, patients often face many other complications, including social impairments and psychiatric comorbidities. Given these considerations, it is essential to expand our understanding of mental disorders in patients with insomnia.

In recent years, most studies concentrated on mental disorders (e.g., anxiety or depression) in patients with insomnia. There is an increasing body of evidence indicating a bidirectional relationship, whereby symptoms of psychiatric disorders lead to insomnia and insomnia also contribute to psychiatric disorders^4,5^. Sleep is essential for brain homeostasis and plasticity; however, sleep disturbances may impair brain plasticity and affect immune and endocrine pathways, thus contributing to psychiatric disorders. Anxiety and depression are the most common psychiatric disorders worldwide, with a global prevalence of 25% and 27%, respectively^2,6^. Moreover, insomnia are common in anxiety or depression; symptoms of insomnia exceed 80% in those who are concurrently depressed or anxious^7^. Anxiety and depression have been shown to have numerous negative effects on patients. These negative influences underscore the importance of improving psychological states. While several studies have focused on describing and quantifying feelings of depression or anxiety among insomnia patients, few have concentrated on reducing anxiety and depression, and whether the effects of these strategies are sustained remains unknown^7,8^.

The network approach is an innovative and burgeoning approach for mathematically analyzing and visually displaying the relationships among multiple variables^9,10^. In network analysis, the variables are represented by nodes, and the connections between the variable are represented by edges (e.g., regularized partial correlations). Therefore, network analysis is a rapidly developing analytical tool that can provide pathways for investigating between-node interactions. According to a network hypothesis, psychological disorders result from the direct causal interactions between the symptoms that constitute them^11^. This suggests that rather than stemming from a single underlying latent variable, psychiatric symptoms may arise from the direct interactions of several symptoms. Moreover, network analysis offers several centrality and predictability indicators (e.g., strengthen) to quantify their importance and controllability in the network. Through the detection of bridging symptoms, it offers some recommendations for the management of comorbidities^12^. Therefore, regarding anxiety, depression, and insomnia as nodes in a network, determining the potential pathways among these nodes is theoretically feasible.

In this study, we constructed a network of sleep-related factors associated with depressive and anxiety symptoms in patients with insomnia. We aimed to find out the core concepts of depression and anxiety in these patients and the interaction between these concepts, with the aim of providing recommendations for improving mental health.

## Method

### Participants

This is an observational study conducted at one of the largest hospitals in Northwestern China, Xijing Hospital, the teaching hospital of the Fourth Military Medical University. Approval for this study was obtained from the ethics committee of Xijing Hospital. All procedures in the present study were designed in accordance with the recommendation of Good Clinical Practice guidelines and the Declaration of Helsinki. All participants provided informed consent. Data was collected between January 1, 2022, and December 31, 2023, through professional outpatient doctor testing.

This study enrolled patients aged 18 to 80 years who met the ICD-10 diagnostic criteria for insomnia. In the stratified analysis, participants were categorized into the following age groups: young adults (18–44 years); middle-aged adults (45–65 years); and older adults (> 65 years). There were no exclusion criteria unless the patients did not agree to participate in this study. The rationale for adopting non-restrictive exclusion criteria was to enhance the generalizability of our findings. While acknowledging the inclusion of secondary insomnia cases (attributable to comorbid conditions or psychotropic medication use), our primary research focus on elucidating the anxiety-depression correlation in insomnia patients justifies the incorporation of a heterogeneous sample, which improves generalizability across diverse clinical subgroups. A total of 1571 patients (678 male, mean age 43.6 ± 14.4 years old) were enrolled. The tests for HAM-A (Hamilton Rating Scale for Anxiety)^13^, HAM-D (Hamilton Rating Scale for Depression)^14^ and PSQI (Pittsburgh Sleep Quality Index)^15^ were done by the same trained and certified neurologist (Zirong Zhu).

### Sleep quality

Sleep quality was assessed using the PSQI, which is the most commonly used generic measure in clinical and research settings for insomnia patients^15^. This is a 19-item self-administered questionnaire that assesses subjective sleep quality during the previous month. A global PSQI score > 5 is associated with poor sleep quality and is determined by evaluating seven components (with subscale scores ranging from 0 to 3): sleep quality, sleep latency, sleep duration, habitual sleep efficiency, sleep disturbance, use of sleeping medication, and daytime dysfunction^16^. The summed scores of seven subscales were of interest to the current study. The internal consistency of PSQI in this study demonstrated good (α = 0.74) level.

### Depression symptoms

We used the HAM-D to assess depression symptoms. The HAM-D, which includes the 17 items (Anxiety /Somatization, Weight, Cognitive disturbance, Diurnal variation, Retardation, Sleep disturbance, Hopelessness) is the version generally used in clinical trials^14,17^. Nine of the HAM-D items adopt the 5-level scoring method from 0 to 4 points, while eight items adopt the 3-level scoring method from 0 to 2 points. A total score of 0–7 is considered to be normal. The internal consistency of the scale in the current study was good (α = 0.82).

### Anxiety symptoms

The Hamilton Rating Scale for Anxiety (HAM-A) measures the severity of anxiety and can also serve as a diagnostic tool^13,18^. The HAM-A includes 14 items rated on a 5-point scale. The total scores of 7 or higher indicate symptoms of anxiety. In this current study, we focus on factor scores, specifically psychic anxiety and somatic anxiety, calculated by dividing the total score of each item in the factor by the number of items in the factorial structure. The scale demonstrated good internal consistency in the current study (α = 0.76).

### Statistical analyses

We used the Gaussian graphical model (GGM) with the R package “qgraph” to estimate the network structure of the network^9,19^. The network approach’s primary benefit is that it makes the data’s multidimensional relationships visually evident. After accounting for the effects of every other node in the network, the edges in a GGM show partial correlations between two nodes. One in which nodes A and B’s connection is the connection after adjusting for every other edge in the network, and edges may be thought of as partial correlation coefficients. In the current study, we used non-parametric test correlation matrices (Spearman correlation coefficient) as input for the estimation of GGM^20^. Then the graphical Least Absolute Shrinkage and Selection Operator (LASSO) algorithm was used to regularize the GGM^21^. This regularization process results in a more stable, easily parsimonious, and sparse network by shrinking all edges and setting edges with modest partial correlations to zero., we set the GGM tuning parameter to 0.5 to reach a good balance between the sensitivity and specificity of determining true edges. We set the Extended Bayesian Information Criterion (EBIC) hyperparameter γ to 0.5 as recommended in previous studies to achieve a reasonable compromise between the sensitivity and specificity of identifying real edges^22^. The Fruchterman–Reingold algorithm (Select “spring” layout) was used to visualize the current network. In the visual representation of the network, blue edges in the network represent positive partial correlations, whereas red edges represent negative partial correlations. There are more partial correlations between the nodes when the edges are thicker.

Recent research showed that strength and bridge expected influence were the most reliable centrality indexes (e.g., betweenness and closeness) for evaluating the importance of nodes in networks^12,23^. Thus, in this study, we calculated these two parameters. The sum of all absolute weight values of the edges that are linked to a node is defined as strength; The sum of the edge weights connecting a given node to all nodes in the other community is defined as bridge expected influence. Strength values indicate the degree of importance in the network, and a higher bridge expected influence value indicates a higher likelihood of activating the opposite community.

The R-package “bootnet” examined the robustness of the network. We calculated the accuracy of edge weights by computing their 95% CI using nonparametric bootstrapping with 1000 samples. Furthermore, the stability of strengthen and bridge expected influence was assessed by calculating the Correlation Stability (CS)-coefficient via a case-dropping bootstrap approach. The ideal CS-coefficient is recommended above 0.5 and should not be below 0.25^24^. The evaluation of whether two edge weights or two node strengths differ significantly from each other was done using bootstrapped difference tests (1000 bootstrap samples) for the edge weights and node strengths.

## Results

### Clinical characteristics

From January 1, 2022, to December 31, 2023, a total of 1571 patients (678 male, mean age 43.6 ± 14.4 years old) with insomnia were enrolled (Table 1). There were 1521 patients with a PSQI score > 5. The prevalence of anxiety (HAM-A > 6) and depression (HAM-D > 7) in the current study was reported to be 87.1% and 88.0%, respectively. Specifically, the mean scores and standard deviations for each scale selected in the present networks were shown in Table 2. The mean PSQI scores of patients were 10.97, which was in accordance with the clinical diagnosis (Table 2).

**Table 1:** Characteristics of the 1571 patients.

Characteristic	Value
Age, year, mean (SD)	43.6 (14.4)
Gender, male, no. (%)	678 (43.2%)
Age groups, no. (%)^a^	
Young adults	815 (51.9)
Middle-aged adults	638 (40.6)
Older adults	118 (7.5)
HAM-A, no. (%)^b^	
None anxiety	203 (12.9)
Possible anxiety	684 (43.5)
Anxiety	485 (30.9)
Obvious anxiety	180 (11.5)
Major anxiety	19 (1.2)
HAM-D, no. (%)^c^	
None depression	189 (12.0)
Mild depression	1086 (69.1)
Moderate depression	294 (18.7)
Major depression	2 (0.1)
PSQI, no. (%)^d^	
Good sleep	50 (3.2)
Mild insomnia	652 (41.5)
Moderate insomnia	764 (48.6)
Major insomnia	105 (6.7)

Data are presented as n (%) or mean (SD).

^a^Young adults (age 18–44); Middle-aged adults (age 45–65); Older adults (> 65).

^b^HAM-A scores ≤ 6 are defined as none anxiety; 7–13 are defined as possible anxiety; 14–20 are defined as anxiety; 21–28 are defined as obvious anxiety; > 28 is defined as major anxiety.

^c^HAM-D scores ≤ 7 are defined as none depression; 8–20 are defined as mild depression; 21–35 are defined as moderate depression; >35 are defined as major depression.

^d^PSQI scores ≤ 5 are defined as good sleep; 6–10 are defined as mild insomnia; 11–15 are defined as moderate insomnia; > 15 are defined as major insomnia.

**Table 2:** Abbreviation, mean scores and standard deviations for each variable selected in the present networks.

Variables	Abbreviation	Value
HAM-A		
Somatic anxiety	A1	0.70 (0.47)
Psychic anxiety	A2	1.18 (1.18)
Total		13.16 (13.16)
HAM-D		
Anxiety/Somatization	B1	5.30 (5.30)
Weight	B2	0.24 (0.24)
Cognitive disturbance	B3	1.62 (1.62)
Diurnal variation	B4	0.44 (0.44)
Retardation	B5	2.02 (2.02)
Sleep disturbance	B6	3.72 (3.72)
Hopelessness	B7	1.52 (1.52)
Total		14.85 (6.32)
PSQI		
Subjective sleep quality	C1	2.38 (0.69)
Sleep latency	C2	2.47 (0.82)
Sleep duration	C3	0.97 (1.03)
Habitual sleep efficiency	C4	0.48 (0.86)
Sleep disturbances	C5	1.40 (0.63)
Use of sleeping medication	C6	1.15 (1.32)
Daytime dysfunction	C7	2.13 (0.94)
Total		10.97 (3.09)

Data are presented as mean (SD).

### Network structure

**Fig. 1:**
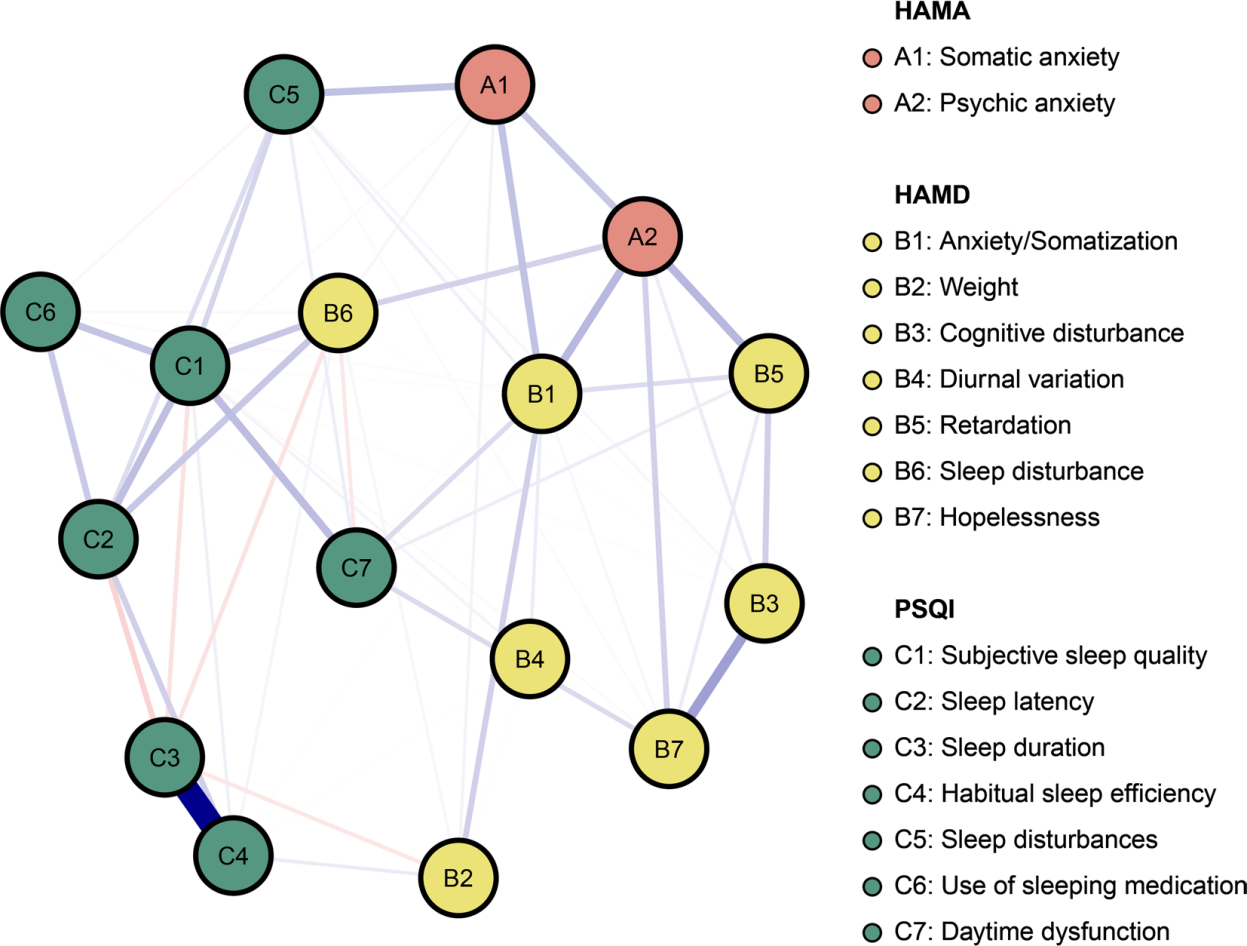
Network structure of anxiety and depression symptoms in insomnia patients. Positive correlations are indicated by blue edges, whereas negative correlations are indicated by red edges. The degree of association is reflected in the edge’s thickness.

**Fig. 2:**
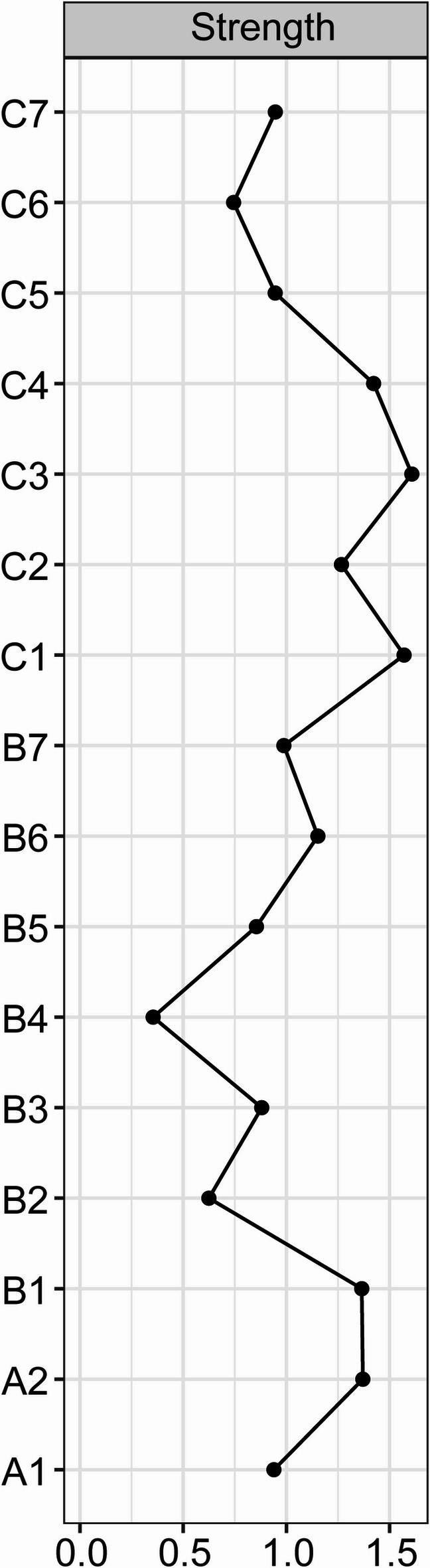
Centrality plot depicting the strength of each variable in this network.

**Fig. 3:**
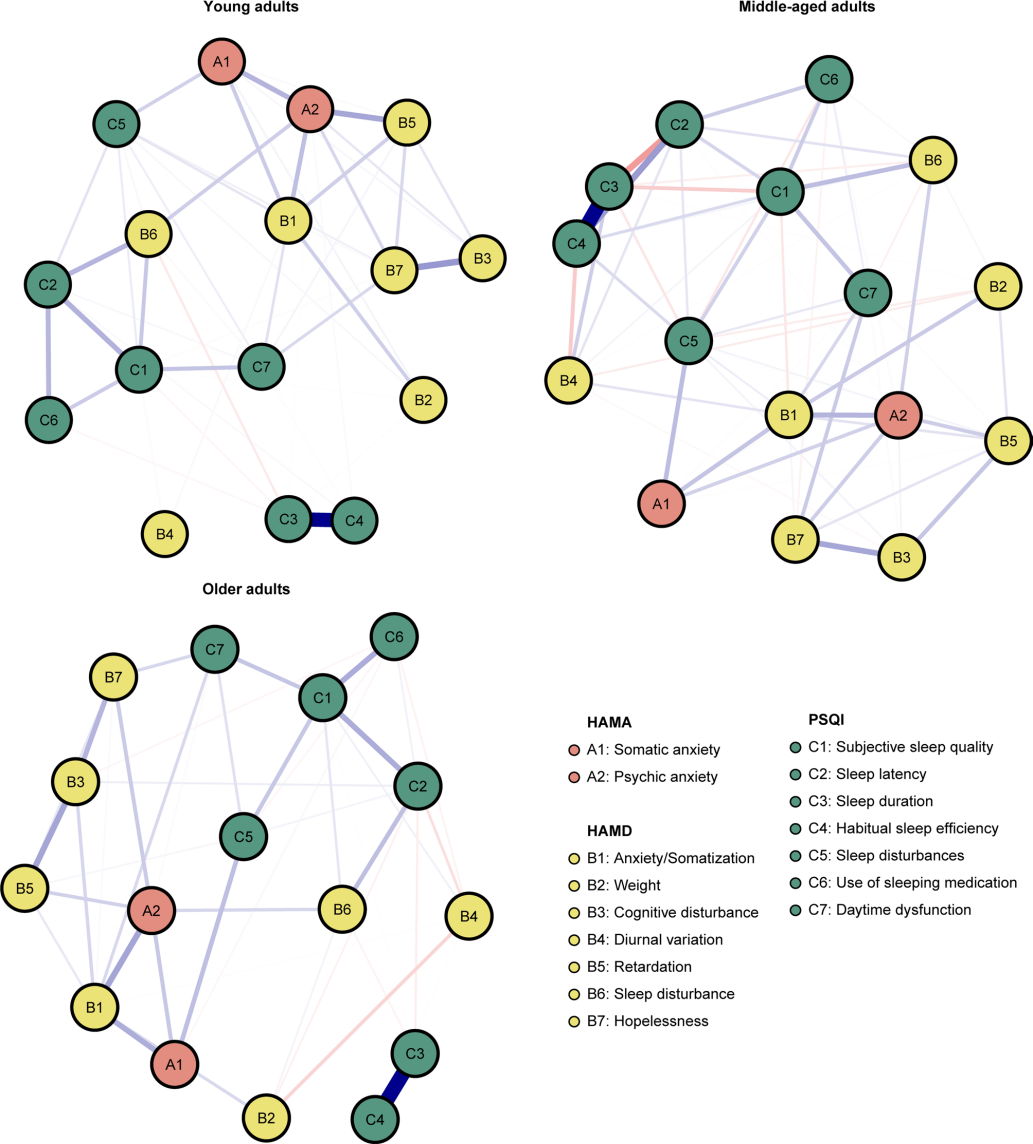
Network structure of anxiety and depression symptoms in insomnia patients across young, middle-aged, and older adults. Young adults (age 18–44); Middle-aged adults (age 45–65); Older adults (> 65).

The network of sleep quality, depressive and anxiety symptoms is shown in Fig. 1. There were several remarkable characteristics in this network. First, there were both positive and negative edges. Second, we found the strongest regularized partial correlation existed between “C3: Sleep duration” and “C4: Habitual sleep efficiency” (*r* = 0.91). The other six strong edges are between “B3: Cognitive disturbance” - “B7: Hopelessness” (*r* = 0.36), “A2: Psychic anxiety” - “B5: Retardation” (*r* = 0.28), “A2: Psychic anxiety” - “B1: Anxiety/Somatization” (*r* = 0.27), “C1: Subjective sleep quality” - “C7: Daytime dysfunction” (*r* = 0.26), “C1: Subjective sleep quality” - “C2: Sleep latency” (*r* = 0.25), and “A1: Somatic anxiety” - “C5: Sleep disturbances” (*r* = 0.24). The five strong negative edges are between “C2: Sleep latency” - “C3: Sleep duration” (*r* = -0.18), “C1: Subjective sleep quality” - “C3: Sleep duration” (*r* = -0.13), “B6: Sleep disturbance” - “C3: Sleep duration” (*r* = -0.13), “B2: Weight” - “C3: Sleep duration” (*r* = -0.11), “B6: Sleep disturbance” - “C7: Daytime dysfunction” (*r* = -0.11). All edge weights within the current network were shown in Table S1. Third, the five items with the greatest strength were “C3: Sleep duration”, “C1: Subjective sleep quality”, “C4: Habitual sleep efficiency”, “A2: Psychic anxiety”, “B1: Anxiety/Somatization” (Fig. 2a). These five concepts were the most associated nodes in this network from a statistical perspective. “B4: Diurnal variation” was the weakest node. Fourth, the bootstrapped 95% confidence intervals of edge weights derived from 1000 bootstrap samples were shown in Fig S1. Fig S2 showed the bootstrapped difference test for edge weights. Fig S3 showed the bootstrapped difference tests for node strength. The CS-coefficient of node bridge strengthen is 0.75, indicating the centrality index (strength) is adequately stable (Fig S4). Fifth, we have divided the patients into three groups based on age and performed stratified analyses within each group. Subgroup analyses among different age strata confirmed the robustness of our findings, demonstrating close alignment with the primary analysis. The strongest regularized partial correlation coefficients also existed between “C3: Sleep duration” and “C4: Habitual sleep efficiency” (Fig. 3, *r* = 0.83, 0.97, and 0.87, respectively).

## Discussion

To our knowledge, this pioneering investigation represents the first network analysis elucidating the network structural interplay between anxiety and depressive symptoms in a large-scale cohort of 1,571 insomnia patients. We investigated the structure of anxiety and depressive symptoms in this sample using network analysis. The prevalence of anxiety and depression was 87.1% (total 1368) and 88.0% (total 1382), respectively, which were much the same in two previous reports from the US and significantly higher than that in the population^2,6,25,26^. We have identified for the first time the core symptoms and their most strongly interconnected manifestations within the anxiety-depressive symptom clusters in sleep disorder patients, providing targeted clinical management recommendations. For underlying mechanisms, it may be interpreted by anomalies in neurobiology, such as hypothalamic-pituitary-adrenal axis activation, serotonin system dysfunction, and overexpression of immune system peptides^27^. This unprecedented focus on symptom network architecture in insomnia patients addresses a research gap, providing clinicians with an evidence-based result for targeted psychological interventions and mood disorder management.

We found the strongest association between the sleep items “C3: Sleep duration” and “C4: Habitual sleep efficiency”. Given that both “C3: Sleep duration” and “C4: Habitual sleep efficiency” were PSQI items, it is understood that the two are closely connected. Although multicollinearity issues remain methodologically contentious, empirical studies have demonstrated that GGM effectively mitigate its distorting effects on network topology^28^. Furthermore, sleep duration and sleep efficiency reflect distinct constructs of objective physiological measurement and subjective sleep efficacy, respectively, both providing specific guidance for developing clinical intervention strategies^7^. Subgroup analyses demonstrated consistent findings in network analyses across different age strata, further substantiating the generalizability of this conclusion. Furthermore, a study conducted during the COVID-19 pandemic in Wuhan among frontline medical staff demonstrated that nightmares were independently correlated with both sleep length and sleep efficiency^29^. In addition to this pair, the other two powerful linkages in this network were “B3: Cognitive disturbance” - “B7: Hopelessness” and “A2: Psychic anxiety” - “B5: Retardation”. Interestingly, the most potent connections between the various scales in this network are “A2: Psychic anxiety” in HAM-A and “B5: Retardation” in HAM-D. This was similar to the findings of Zhou et al. with major depression, which indicated that the depressed mood directly triggered psychological anxiety and retardation^30^. In other words, there is a strong positive correlation between psychic anxiety and retardation, even if the participants are different. Considering all of the above, these associations might be points of intervention in improving the mental health level of sleep disorder patients.

Among all the items included in this current network, “C3: Sleep duration” has the highest strength centrality, followed by “C1: Subjective sleep quality”, “C4: Habitual sleep efficiency”, “A2: Psychic anxiety”, “B1: Anxiety/Somatization”, and “B4: Diurnal variation” has the lowest strength centrality. The top three items belong to sleep symptoms. Some studies indicate that focusing on these nodes might have the greatest overall impact on other nodes within the network due to their strong centrality^31^. We believe that sleep quality is at the core of this current network. Tao and coworkers investigated the symptoms of anxiety, depression, and sleep disturbance among college students during the COVID-19 pandemic and found a similar outcome to our study^32^. Sleep dissatisfaction is the strongest centrality symptoms in that network. Therefore, sleep duration is crucial for both medication-regulating treatment and promoting mood improvement in patients with insomnia.

However, insomnia constitute a heterogeneous disease entity encompassing primary, comorbid, and substance-induced subtypes^1^. Each subtype manifests distinct pathophysiological mechanisms and clinical characteristics. Differential symptom connectivity patterns may emerge across subtypes through specific biopsychosocial mechanisms^11^. Notably, the most central symptoms and strongest connecting nodes in the network may also vary across different types of insomnia disorders. Current evidences suggests that symptom nodes with bridge effects in this network model may demonstrate relative stability^33,34^. The findings substantiate that subjective sleep quality perception and objective sleep duration measurement remain core driving factors for anxiety-depression comorbidity in insomnia patients. Subsequent investigations should implement large-scale longitudinal investigations targeting specific clinical subpopulations.

In fact, some interventions have been applied to alleviate these symptoms^35,36^. Over the past 20 years, CBT-I and medications has been recommended for the treatment of insomnia by most of the regulatory agencies and professional societies^1^. Shaffer found that CBT-I may help improve sleeping and prevent the development psychological distress in patients with insomnia^35^. More recently, a study to explore the efficacy of CBT-I and medications (e.g., zolpidem) for improving daytime functions among patients with insomnia provided sufficient evidence. Improvements in insomnia, anxiety, and depressive symptoms were observed with CBT and zolpidem, and there were no differences between treatments^36^. In conclusion, it might be regarded as a crucial therapeutic target to help individuals increase the quality of sleep rather than target anxiety and depressive symptoms. Therapeutically addressing these bridging symptoms may be helpful for doctors in treating or preventing comorbidities.

Despite the novelty of the present findings, there are some limitations to our study. First, we are unable to determine the direction of the edges because this study was conducted using cross-sectional data. It is unclear whether the most central node initiates other nodes, is triggered by other nodes, or does both. Future research should implement systematic longitudinal monitoring of outpatient cohorts through structured follow-up assessments conducted at predefined intervals (e.g., 3-month and even 6-month), integrated with standardized psychometric evaluations. Subsequent application of cross-lagged panel modeling (CLPM) would enable rigorous examination of bidirectional causal relationships among symptom clusters, thereby informing the development of data-driven precision intervention strategies. Second, there are 50 (3.2%) people who couldn’t diagnose insomnia based on the PSQI questionnaire. These patients mainly complained that their sleep distribution interfered with their daily lives, despite having a PSQI score of five or lower. We think these patients can all be categorized as having insomnia. Therefore, the finding also has considerable universality. Third, we did not examine the various networks underlying the various forms of insomnia. In general, there are three types of insomnia: primary insomnia, insomnia related to a medical or mental disease and insomnia related to the intake or abuse/dependency from substances. As different types of insomnia may have different levels of anxiety and depressive symptoms, even different works, more work is needed to answer these questions. Fourth, the current analytical framework, predominantly based on assessment instruments including the HAM-A, HAM-D, and PSQI, has not comprehensively incorporated other clinically relevant dimensions such as stress reactivity, interpersonal dysfunction, and cognitive emotion regulation deficits. This selective inclusion may compromise the validity of the symptom network model and constrain the generalizability of research findings. Subsequent investigations will incorporate multidimensional assessment tools to develop a more comprehensive symptom network model.

## Conclusion

In summary, our study offers a new perspective on the complexity and richness of the connections involved in the structure of anxiety and depression in insomnia patients. We highlighted the bridge roles “C3: Sleep duration”, “C1: Subjective sleep quality”, “C4: Habitual sleep efficiency”, “A2: Psychic anxiety”, “B1: Anxiety/Somatization”. Targeting intervention for these crucial symptoms may produce significant benefits. Our findings may offer some help for the clinical prevention and intervention of insomnia patients. When managing patients with insomnia comorbid with anxiety and depressive symptoms, clinicians should prioritize improving sleep duration. Not all such cases necessitate immediate initiation of anxiolytic or antidepressant pharmacotherapy. Enhancement of sleep quality often leads to significant alleviation of concomitant anxiety and depressive symptoms. Ultimately, the aforementioned conclusions necessitate validation through further large-scale studies with expanded sample sizes.

## Electronic supplementary material

Below is the link to the electronic supplementary material.

Supplementary Material 1

## Data Availability

All the experiment data and code used in the analysis procedure are available from the corresponding author upon reasonable request due to that there is a need for a formal data sharing agreement.
