# Supplementary material for: The relationship between anxiety and depression symptoms in insomnia patients: a network analysis
Source: Sci Rep. 2025 Jul 11;15:25030. doi: 10.1038/s41598-025-09746-w (PMC12254401; doi:10.1038/s41598-025-09746-w)
Supplement: Supplementary file 1 — Supplementary Material 1 [file 41598_2025_9746_MOESM1_ESM.pdf]

Supplementary Materials

Table S1. All edges weights within the current network.

Figure S1. Edge-weights accuracy.

Figure S2. Bootstrapped difference test for edge weights.

Figure S3. Bootstrapped difference test for node strengths.

Figure S4. Stability of node strength.

Table S1. All edges weights within the current network.

	A1	A2	B1	B2	B3	B4	B5	B6	B7	C1	C2	C3	C4	C5	C6	C7
**A1**	1	0.61	0.61	0.32	0.38	0.09	0.43	0.33	0.41	0.22	0.22	-0.17	-0.08	0.50	0.07	0.33
**A2**	0.61	1	0.69	0.32	0.52	0.10	0.65	0.42	0.58	0.30	0.28	-0.16	-0.04	0.45	0.08	0.43
**B1**	0.61	0.69	1	0.39	0.46	0.16	0.58	0.3	0.51	0.21	0.21	-0.12	-0.02	0.45	0.02	0.45
**B2**	0.32	0.32	0.39	1	0.20	0.02	0.26	0.25	0.25	0.17	0.16	-0.16	-0.05	0.20	0.10	0.20
**B3**	0.38	0.52	0.46	0.20	1	0.04	0.53	0.15	0.61	0.13	0.16	-0.02	0.03	0.34	-0.02	0.31
**B4**	0.09	0.1	0.16	0.02	0.04	1	0.11	0.100	0.06	0.13	0.09	-0.03	-0.05	0.09	-0.01	0.08
**B5**	0.43	0.65	0.58	0.26	0.53	0.11	1	0.24	0.53	0.19	0.19	-0.08	-0.02	0.37	0.04	0.41
**B6**	0.33	0.42	0.3	0.25	0.15	0.10	0.24	1	0.20	0.51	0.51	-0.35	-0.19	0.33	0.32	0.18
**B7**	0.41	0.58	0.51	0.25	0.61	0.06	0.53	0.20	1	0.15	0.14	-0.07	0.00	0.36	0.04	0.42
**C1**	0.22	0.30	0.21	0.17	0.13	0.13	0.19	0.51	0.15	1	0.58	-0.32	-0.15	0.41	0.45	0.39
**C2**	0.22	0.28	0.21	0.16	0.16	0.09	0.19	0.51	0.14	0.58	1	-0.26	-0.06	0.39	0.43	0.27

**C3**	-0.17	-0.16	-0.12	-0.16	-0.02	-0.03	-0.08	-0.35	-0.07	-0.32	-0.26	1	0.92	-0.15	-0.23	-0.05
**C4**	-0.08	-0.04	-0.02	-0.05	0.03	-0.05	-0.02	-0.19	0.00	-0.15	-0.06	0.92	1	-0.04	-0.11	0.01
**C5**	0.50	0.45	0.45	0.20	0.34	0.09	0.37	0.33	0.36	0.41	0.39	-0.15	-0.04	1	0.12	0.39
**C6**	0.07	0.08	0.02	0.10	-0.02	-0.01	0.04	0.32	0.04	0.45	0.43	-0.23	-0.11	0.12	1	0.15
**C7**	0.33	0.43	0.45	0.20	0.31	0.08	0.41	0.18	0.42	0.39	0.27	-0.05	0.01	0.39	0.15	1

A = Hamilton Rating Scale for Anxiety, B = Hamilton Rating Scale for Depression, C = Pittsburgh Sleep Quality Index.

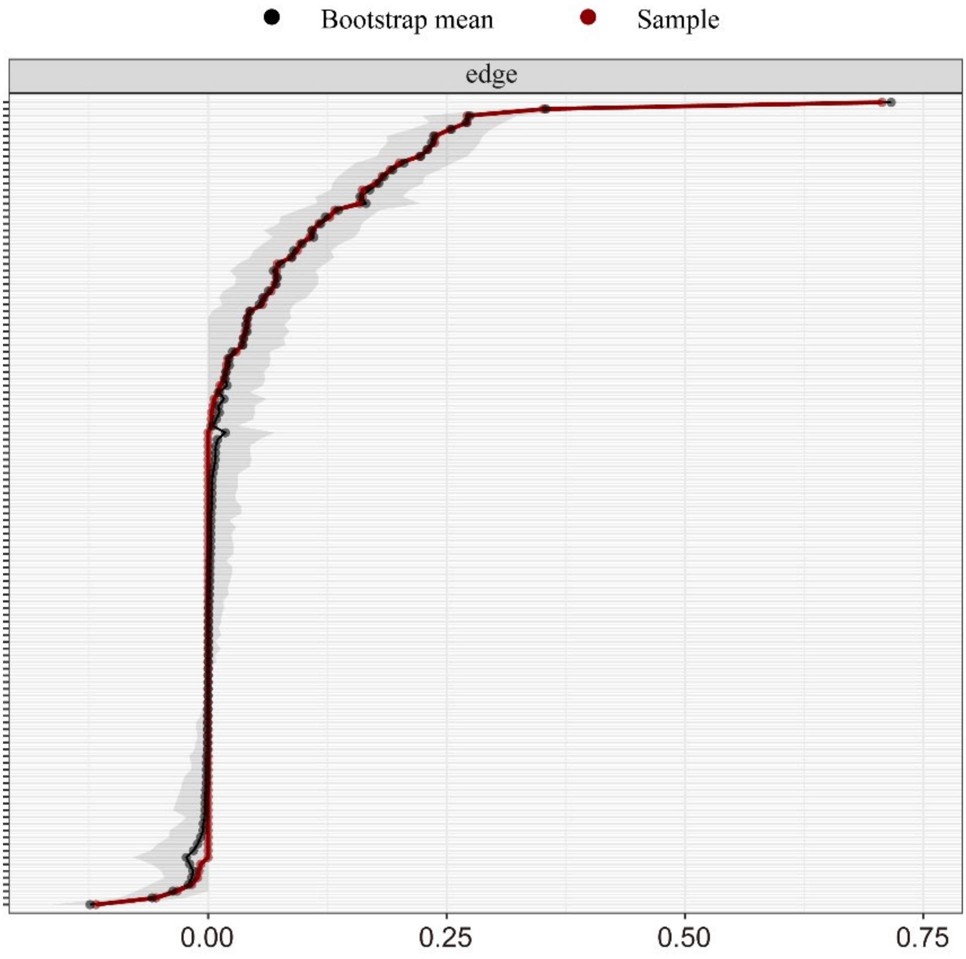

Figure S1. Edge-weights accuracy. The red line indicates the edge weight values and the gray area the 95% CIs.

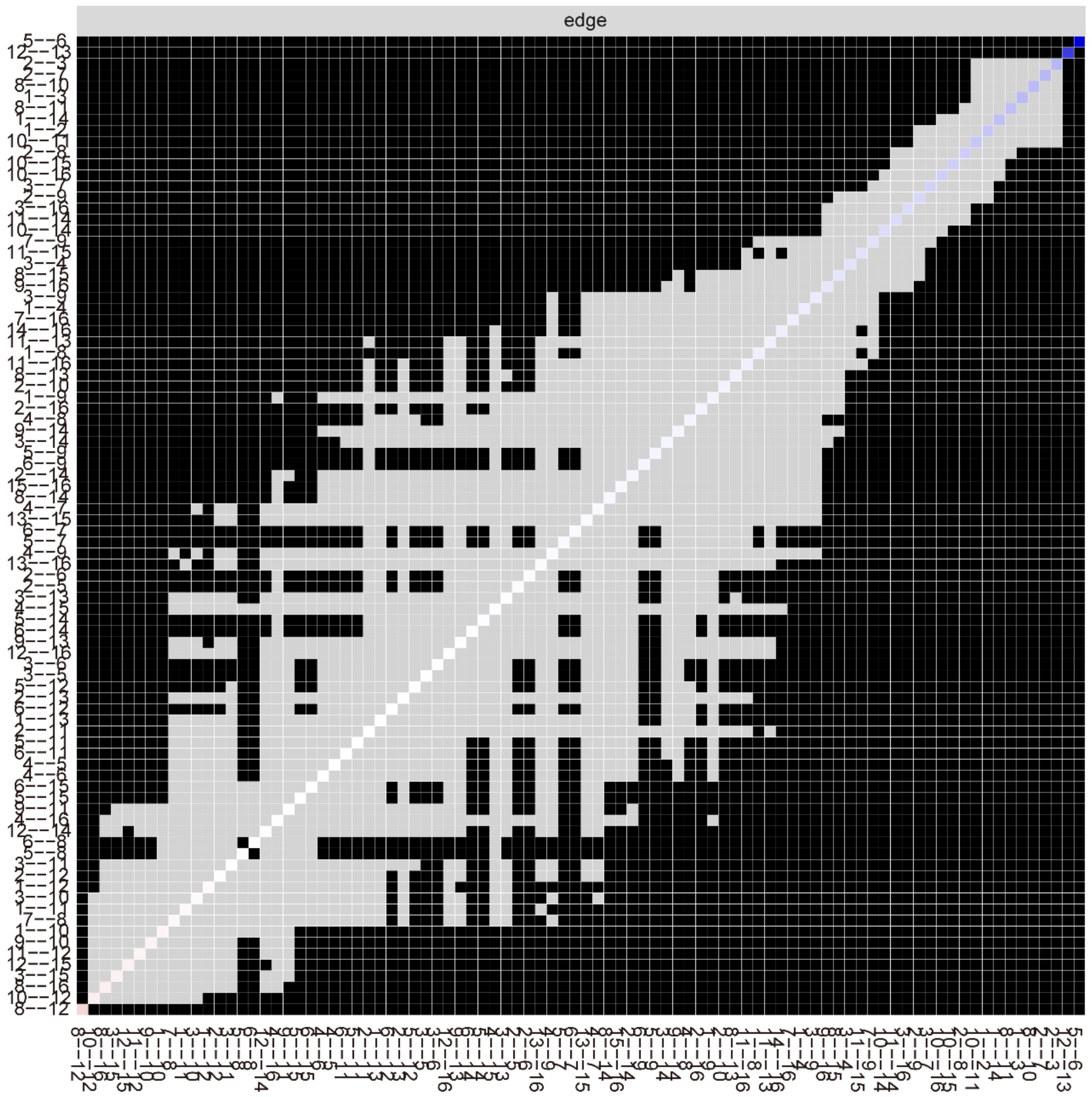

Figure S2. Bootstrapped difference test for edge weights. Gray boxes indicate edge weights that do not differ significantly from one another, while black boxes indicate edge weights that do differ significantly. Blue and red boxes on the diagonal correspond to edge weights with positive and negative correlations, respectively.

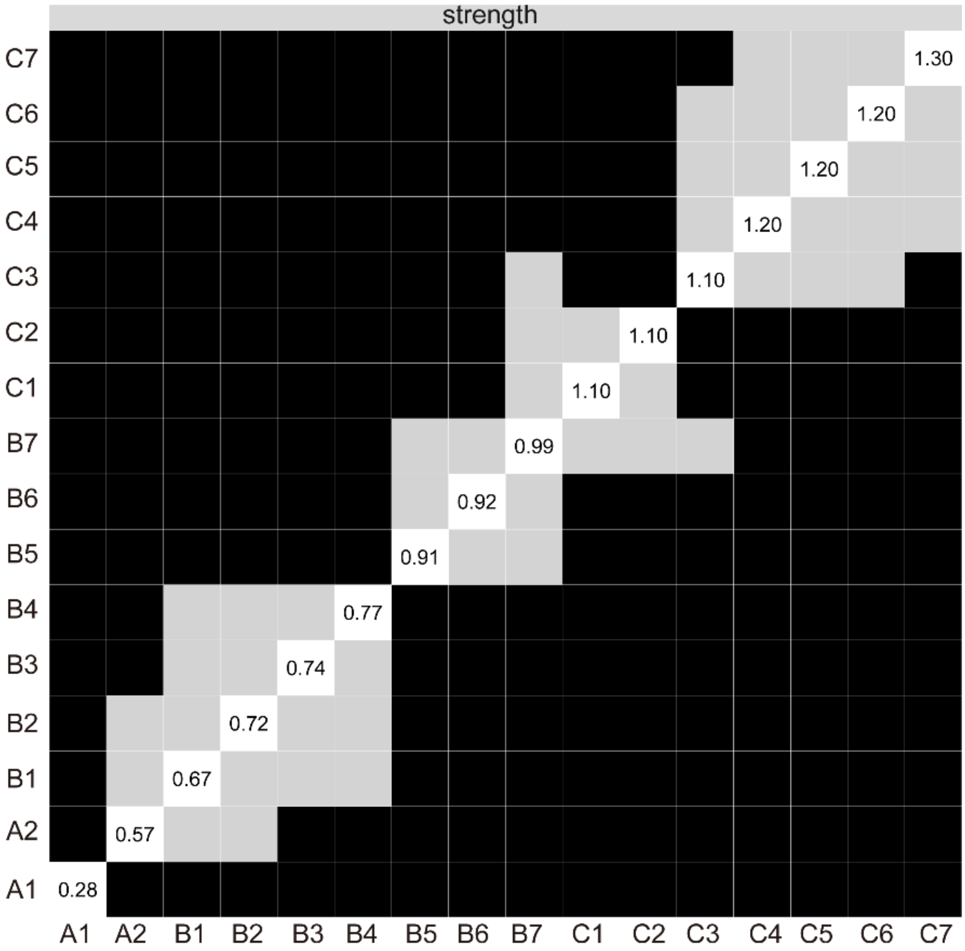

Figure S3. Bootstrapped difference test for node strengths. Gray boxes indicate node strengths that do not differ significantly from one another, while black boxes indicate node strengths that do differ significantly. The number in the white boxes represent the value of node strengths.

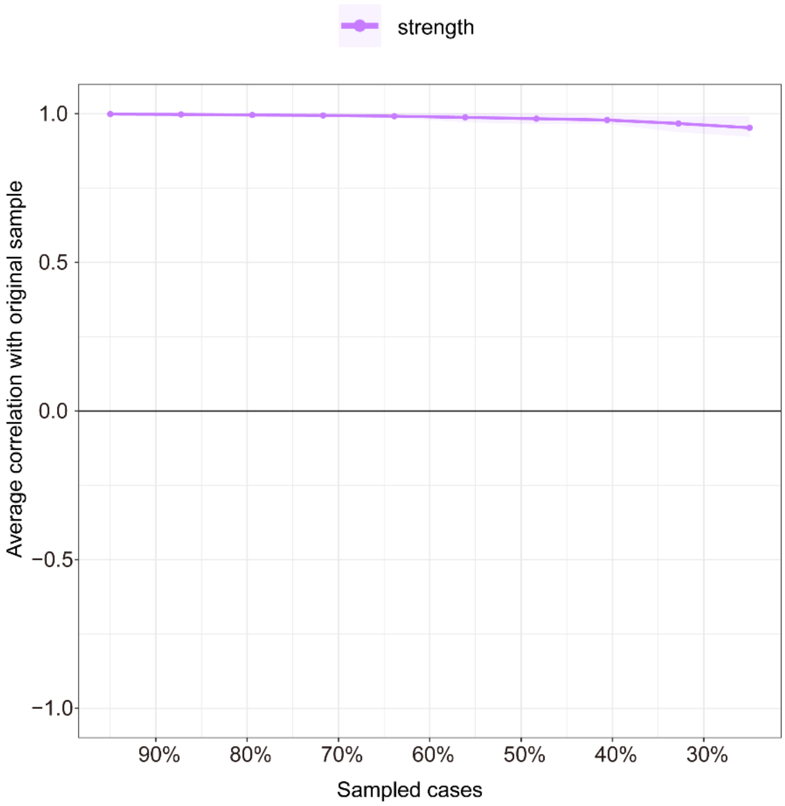

Figure S4. Stability of node strengths and bridge expected influence. The purple bar represents the average correlation between strength in the full sample and subsample with the purple area depicting the 2.5th quantile to the 97.5th quantile.
